# Elastic Modulus and Flatwise (Through-Thickness) Tensile Strength of Continuous Carbon Fibre Reinforced 3D Printed Polymer Composites

**DOI:** 10.3390/ma15031002

**Published:** 2022-01-27

**Authors:** Khalid Saeed, Alistair McIlhagger, Eileen Harkin-Jones, Cormac McGarrigle, Dorian Dixon, Edward Archer

**Affiliations:** 1 Engineering Research Institute, Ulster University, Jordanstown Campus, Newtownabbey BT37 0QB, UK; a.mcihagger@ulster.ac.uk (A.M.); e.harkin-jones@ulster.ac.uk (E.H.-J.); d.dixon@ulster.ac.uk (D.D.); 2 School of Computing, Engineering and Intelligent Systems, Ulster University, Magee Campus, Londonderry BT48 7JL, UK; c.mcgarrigle1@ulster.ac.uk

**Keywords:** additive manufacturing, 3D printing, carbon fibre, polymer composites, through-thickness, computed tomography

## Abstract

Additively manufactured composite specimens exhibit anisotropic properties, meaning that the elastic response changes with respect to orientation. Both in-plane and out-of-plane mechanical properties are important for designing purpose. Recent studies have characterised the in-plane performance. In this study, however, through-thickness tensile strength of 3D polymer composites were determined by printing of continuous carbon fibre reinforced thermoplastic polyamide-based composite, manufactured using a Markforged Two 3D printer. This paper discusses sample fabrication and geometry, adhesive used, and testing procedure. Test standards used to determine out-of-plane properties are tedious as most of the premature failures occur between the specimens and the tabs. Two types of samples were printed according to ASTM flatwise tension standard and the results were compared to determine the geometry effect on the interlaminar strength. This test method consists of subjecting the printed sample to a uniaxial tensile force normal to the plane. With this method, the acceptable failure modes for tensile strength must be internal to the structure, not between the sample and the end tabs. Micro-computed tomography (µCT) was carried out to observe the porosity. Surface behaviour was studied using scanning electron microscopy (SEM) to see the voids and the distribution of the fibres in the samples. The results showed consistent values for tensile strength and elastic modulus for Araldite glue after initial trials (with some other adhesives) to determine a suitable choice of adhesive for bonding the samples with the tabs. Circular specimens have higher tensile strength and elastic modulus as compared to rectangular specimens.

## 1. Introduction

Fused deposition modelling (FDM) is one of the most widely used 3D printing technologies for fabricating complex geometry with the capacity to compete in strength and stiffness with conventional processing techniques [1]. In the FDM technique, the polymer filament is extruded through a heated nozzle that traces the part cross-sectional geometry layer by layer. Thermoplastic polymers are the most frequently used feed-stock materials for FDM due to their relative low cost as well as low melting temperature. Due to the low strength for functional engineering parts, the use of thermoplastic polymer is low [2]. This issue has been addressed by researchers by adding reinforcement such as fibre to the polymer matrix to produce a composite structure with improved mechanical properties [3]. Reinforcing fibre can be either short fibre or continuous fibre; polymer composites fabricated using short fibres are attractive because of its economical and superior mechanical properties compared to pure thermoplastic polymer. As the short fibre increases the strength marginally, researchers are moving towards incorporating continuous fibre in the matrix material to transfer load between the fibres. Currently, polymer composites reinforced with continuous fibre are one of the largest focus areas in 3D printing research due to its exceptional mechanical properties [4], but in all the methods examined, the fibres are laid in the in-plane x-y orientation, with no reinforcing fibres in the z-direction.

Fibre-based polymer composites are increasing for several applications in the transportation industry, construction industry, and in the medical field, as they are lightweight with favourable strength and stiffness properties. Normally, the fibres in composite materials are positioned so that it can resist the applied loads in the printing direction. Normal stresses developed from axial loads are resisted by the resin and fibre. Several researchers studied the in-plane mechanical properties (tensile, flexural) using continuous reinforced polymer composites [5]. In-plane test standards are developed for testing composite materials in tension or compression. Saeed et al. [6] studied the in-plane mechanical properties of thermoplastic polymer composites reinforced with continuous carbon fibre samples that were fabricated using a Mark Forged Two 3D printer. Strength and elastic modulus were measured using a series of tensile tests and the effect of fibre orientation on the in-plane mechanical properties were evaluated. Tensile strength of 604 MPa and elastic modulus of 73 GPa were measured for unidirectional samples with 29% fibre volume fraction. Recently, Saeed et al. [7] examined the effect of post-processing on 3D printed polymer composites and it was noticed that strength and elastic modulus increased significantly by 29% and 11%, respectively, due to the reduction of voids and the increase of fibre volume fraction by 6%. From the micro sectioning analysis, it was confirmed that the void contents were reduced from 3.96% to 0.26%. Caminero et al. [8] studied the impact damage resistance of 3D printed thermoplastic composites fabricated using fused deposition modelling. However, out of plane properties are not often considered in designing a structure. Currently, there are very limited number of studies on developing the test method, sample designing, and test result analysis of 3D printed polymer composites to measure strength and modulus. One of the greatest limitations of continuous fibre filament reinforcement is the failure to link the layers printed on the x-y plane to the 3rd direction as no pressure is applied during the printing process; pressure plays a vital role and is directly related to the increase of gap (pores) between the layers. To evaluate the performance in the z-direction, through-thickness testing was performed to evaluate the strength in the z-direction. Through-thickness (flatwise tensile strength) is a mechanical assessment property in which a tensile force is applied perpendicular to the reinforcement plane. The main purpose of this type of force is to measure the resistance to lamellar tearing which is notoriously weaker in laminar composite materials. Isam et al. [9] studied the through-thickness properties of the samples fabricated using the additive manufacturing (AM) technique by reinforcing nylon with continuous carbon fibre. Fibre volume fraction was varied to study the effect on the interlaminar property of the composite samples. Marco et al. [10] worked on the modified test geometry and test setup to measure the through-thickness tensile strength. Experimentally measured failure load was validated numerically using Abaqus by the application Puck’s 3D failure criteria. In many applications, such as lugs and the hull of the ship, proper attention is needed, as failure normally occurs due to delamination and out of plane properties; therefore, through-thickness design allowables are required. Delamination damage in composite materials has been widely studied because of the weakness in the out-of-plane direction, but little work had been conducted on 3D printed composites. 

Zhang et al. [11] studied the misalignment of fibre that formed during fabrication process and breakage of fibre in polymer composites using reinforced continuous carbon fibre in 3D printing. It was noticed that with the increase in turning angle and curvature, lots of printing defects and aggravate occurred due to the excessive tensile force from the nozzle. In addition, the increase in fibre volume fraction had a great influence on the defect formation. Chen et al. [12] investigated the effect of carbon fibre reinforcement on mechanical performance and negative Poisson’s ratio. The results showed that the effective elastic modulus and compression strength of the composite specimen could be enhanced almost linearly by adding a small amount (0.23%) of continuous carbon fibre up to 59% with bigger negative Poisson’s ratio. Yu et al. [13] conducted the study to examine the anisotropic microstructure of 3D printed composites reinforced with short carbon fibre to determine the effect of anisotropy on mechanical properties. It was evident from the experiments that the microstructural anisotropy significantly varied depending on the printing direction. 

This paper focuses on the fabrication of the specimens and the testing procedure that are employed in conducting the experimental investigation to evaluate the elastic modulus and the through-thickness strength (interlaminar) of the parts manufactured using FDM technique. AM is the latest manufacturing process added which has its own design opportunities and challenges. Therefore, it is necessary to evaluate the properties by applying standard test methods and procedure. Information regarding the equipment used, test fixture, and the bonding of the specimen to the tabs are also discussed. The main aim of this work is to evaluate the properties in the z-direction; a lot of data is available on the in-plane mechanical properties (x and y-direction) of 3D printed polymer composites, but further work is required to determine and understand the weaker out-of-plane response to assist designers in modelling using numerical tools and allow for safe component design.

## 2. Materials and Methods (Specimen Design) 

**Figure 1:**
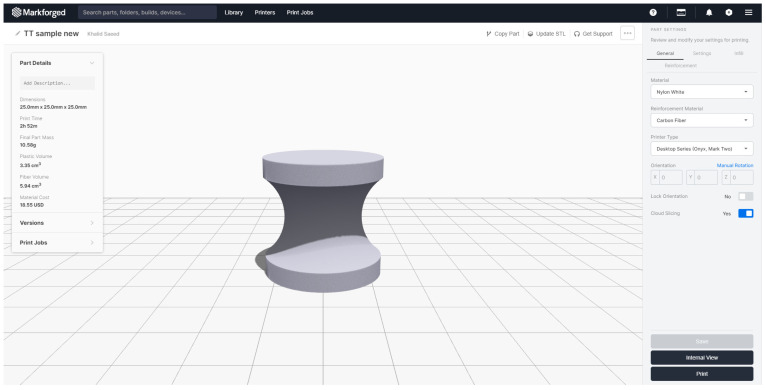
Sample design and the amount of polymer and fibre calculated by the slicing software (Eiger).

**Figure 2:**
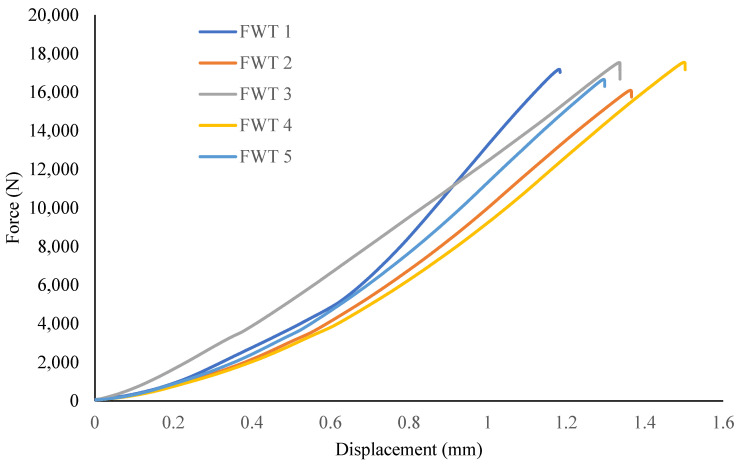
Load versus displacement curve for through-thickness specimens.

Test samples were fabricated according to ASTM standards [14] for through-thickness testing, with a diameter of 25 mm and a 7 mm nominal height, as shown in Figure 1, and were tested using an Instron machine (model 5500R, Norwood, MA, USA), with a 100 kN load capacity. Before printing, the samples were sliced in Eiger software available with the 3D printer, which also gave the estimate of the volume of fibre and polymer. At least five specimens per test condition were used unless valid results were obtained with fewer specimens. The test specimens used were square and circular cross-sections with the end section equal to the thickness of the bonding panel. According to the standard, the coupons are cylindrical or a reduced gage section. The nominal diameter where the specimen is bonded to the metal end tabs is 25 mm, and it may vary from 20 mm to 28 mm according to the standard. The reinforcement fibre-filled type was selected as isotropic for each layer of fibre, with top and bottom layers composed of nylon, which was removed before mounting to the tabs with the adhesive. End tabs were machined from steel bars using a lathe and were bonded with the specimens using jigs to align the parts. Specimens were cured at room temperature for a minimum of twenty-four hours prior to testing so the glue/adhesive could reach its maximum efficiency. After this, the specimens were mounted with the strain gages to measure the elastic modulus accurately. Figure 2 shows the load verses displacement curves for five through-thickness samples with consistent values. 

### Through Thickness Testing of Polymer Composites (Test Method)

In polymer-based composites fabricated using FDM technique, fibre reinforcement happens in a plain-wise fashion, that is, a layer-by-layer format, which exhibits anisotropic properties [15]. This causes the relatively low structural strength of the composite, as well as relative weak fibre–matrix interface between layers, which can be assessed by a through-thickness test (also known as z-direction tensile testing). In this method, force is applied to a test specimen whose axis is perpendicular to the reinforcement plane of a composite specimen. The through-thickness test method is fully developed for polymer composites fabricated through conventional methods, and specific standards are in use for testing through-thickness direction. However, very little literature is available for specimens manufactured through AM techniques. 

The through-thickness test of the 3D printed samples were carried out using a tensile machine with a test speed of 0.5 mm/min. Two strain gages were mounted to the centre of the specimens to measure average strain. Using the elastic region of the stress-strain curve, the elastic modulus was calculated. Several factors that influenced the through-thickness tests were bonding (facing) material, adhesive type, samples fabrication technique, specimen geometry, specimen alignment, and speed of testing. The Araldite two-part epoxy was considered suitable for this test type as it provides high mechanical properties along with excellent environmental resistance. Prior to bonding, the composite was dried, and the bonding surfaces wiped with a suitable cleaning solvent that did not affect the surfaces physically or chemically. Initially, some trial tests were conducted using different speeds until the ultimate strength of the material and the compliance of the system were known. The standard head displacement rate was 0.50 mm/min, which was kept constant for all the test specimens. 

## 3. Results and Discussion

Through-thickness (flatwise) tests were carried out to determine the strength for each type of configuration. Initially the value of stress achieved was 7 MPa for the non-fracture samples as the failure did not in the gage length of the specimen. Several factors could be involved in the test failure due to the debonding between the sample and the steel tabs. Firstly, it might be because of the adhesive, as it might not be cured fully before testing, or it had not reached the ultimate strength. Secondly, the surface of the specimens might have not been roughened enough to provide a better surface area for bonding. After this, the adhesive was changed and the surface was roughened more deeply, as nylon is notoriously difficult to bond due to presence of slip additives within the material, which make it difficult for the adhesive to bond properly the nylon surface, thus causing premature failure (delamination) in the glue and nylon surface. For better adhesion, the outer layers of nylon were removed, and the tabs were bonded to the fibre-reinforced layers which gave more rough area for bonding, and it caused the failure in the desired area. Environmental conditions were kept in mind, as they might affect the tabs chemically if not selected properly. 

**Figure 3:**
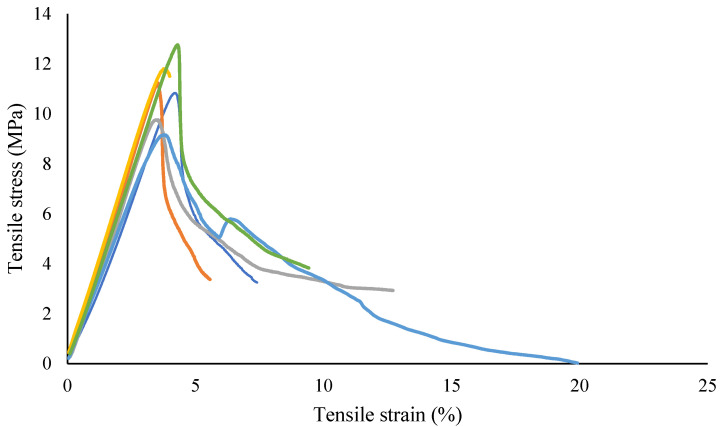
Tensile stress versus strain (displacement) behaviour for circular geometry samples.

**Figure 4:**
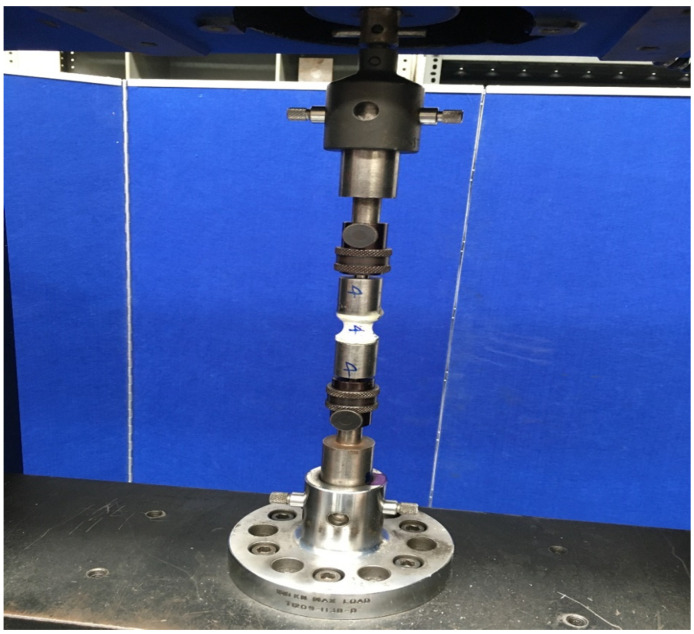
Through-thickness sample during testing in the tensile machine.

**Figure 5:**
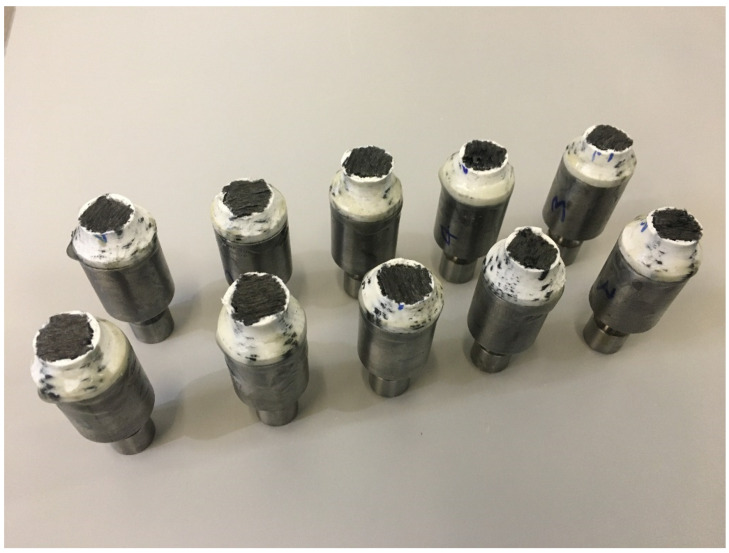
Fracture samples after testing with a consistent failure region.

**Figure 6:**
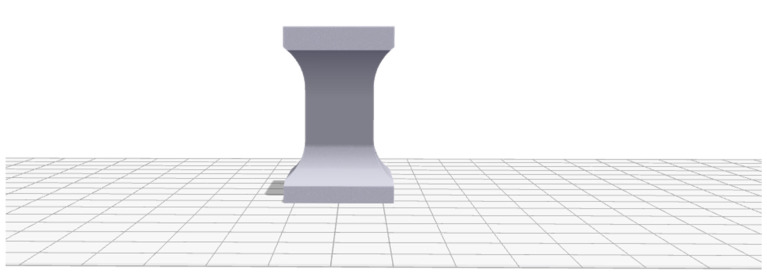
Rectangular through-thickness sample for measuring tensile strength and elastic modulus.

**Figure 7:**
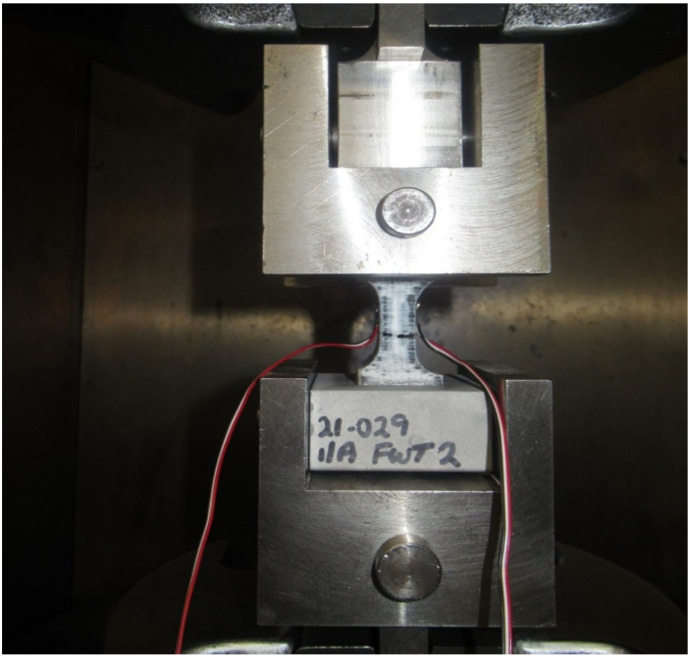
Sample test setup to calculate elastic modulus with two strain gauges.

As in these tests, the samples were subjected to uniaxial tensile force that was normal to the plane of the composite material. Figure 3 shows the stress-strain behavior for circular sample, while Figure 4 shows the test setup. Dimensions of the sample were recorded before each test. It is evident from Table 1 that the Mark Forged Two 3D printer has great accuracy and precision which is due to the stiffness added by the carbon fibre to the printed part. Failure modes acceptable in the flatwise test are those which are internal to the structure as shown in Figure 5, which vary up to 2 mm from the middle of the specimen. Ultimate through-thickness tensile strength was calculated using $F=\frac{P_{max}}{A}$. For rectangular samples (Figure 6), four strain gages can be used on all four faces to achieve better accuracy. A maximum value for tensile strength of 12 MPa and an elastic modulus value of 2.94 GPa were measured for circular specimens; for rectangular specimens, a tensile strength of 7.8 MPa and an elastic modulus of 2.8 GPa were obtained. The maximum tensile strength obtained is 7 MPa, determined by Isam et al. [9]. Two strain gages (Figure 7) were mounted on both circular and rectangular specimens, and elastic modulus was calculated from the data obtained from strain gages and the Instron machine. 

Porosity affects the strength and stiffness of the fibre composite material, but it is difficult to measure its contribution due to other parameter effects, like printing speed, infill density and pattern, and temperature, which can be measured or characterized using experimental techniques. The increase in the porosity content in the AM technique is directly proportional to the size of the reinforcement. However, the increase of fibre content improves the dimensional stability of the printed part through minimizing shrinkage and distortion [16].

Another standard that is used to determine the strength and modulus is ASTM C 297. Strain gauges were bonded to the specimen to measure the elastic modulus correctly. 

**Figure 8:**
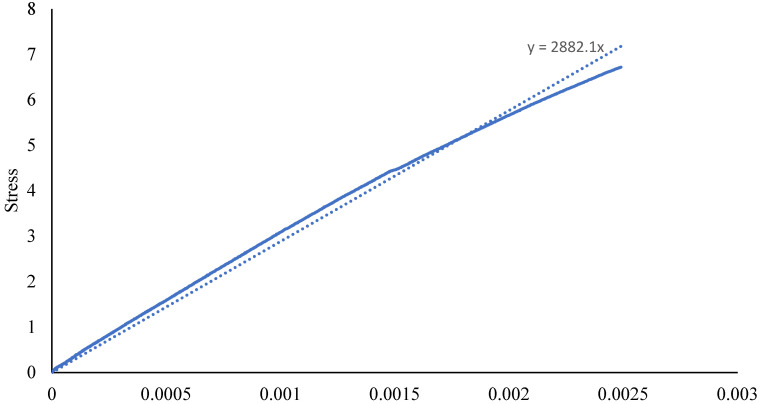
Elastic modulus measurement from strain gages data.

Figure 8 shows the stress and strain data which were obtained from the testing equipment (Instron) and the stain gages, respectively, and which were used to calculate the elastic modulus using the linear part; the mean value of 2.94 GPa was obtained for the rectangular sample, while for circular sample, it was 2.88 GPa. Stress data were obtained from the machine, while the strain data were obtained from two strain gages, which were synchronized with each other as the data were obtained from two different systems.

## 4. Scanning Electron Microscopy

**Figure 9:**
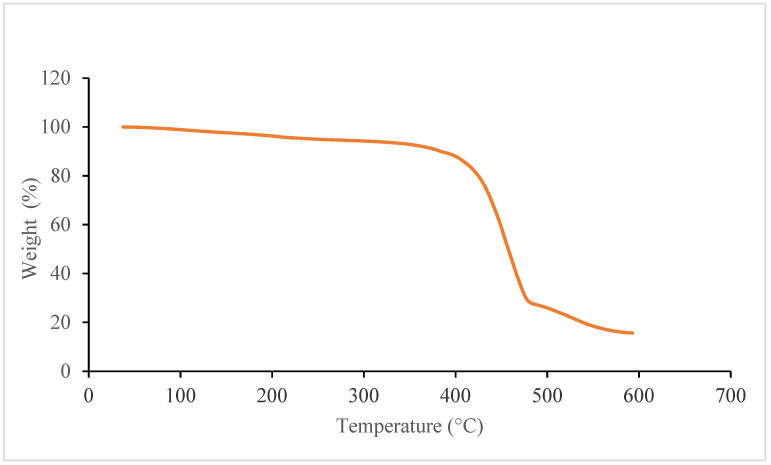
Thermogravimetric analysis (TGA) of polymer composites to obtain fibre for scanning electron microscopy (SEM) analysis.

**Figure 10:**
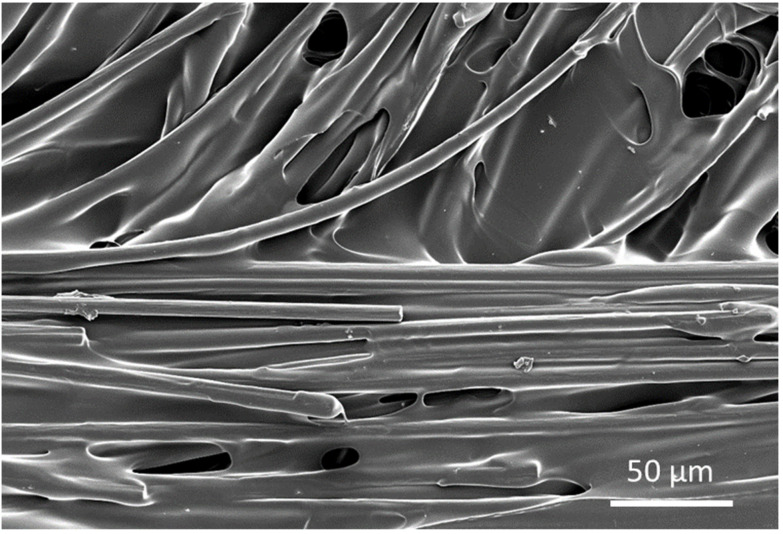
SEM analysis showing fibre distribution with resin, along with voids between deposited filaments.

**Figure 11:**
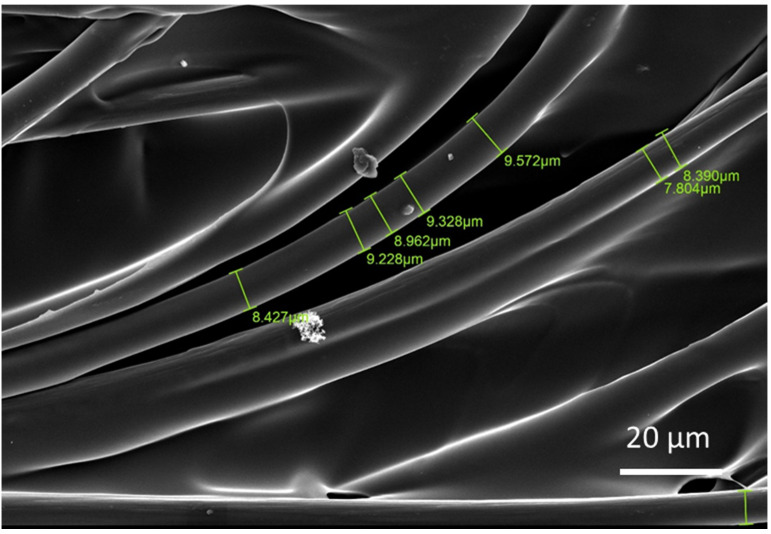
SEM micrograph of through-thickness samples showing fibre diameter and voids.

**Figure 12:**
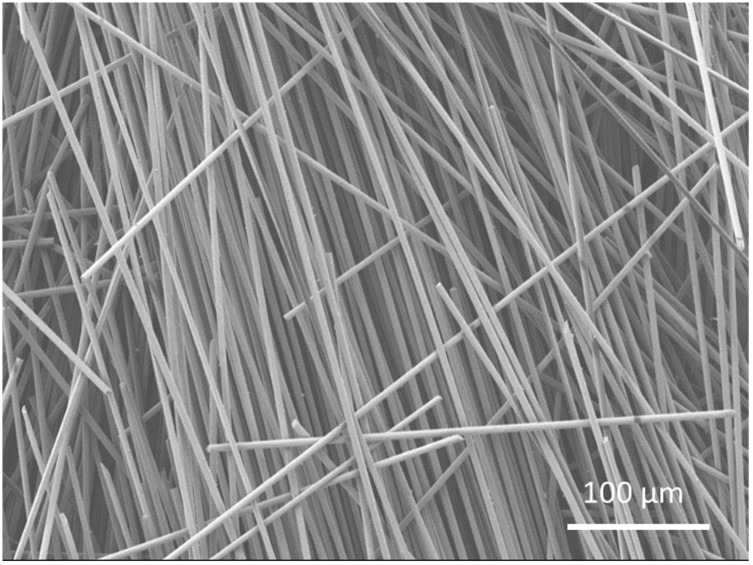
SEM image of the fibres obtained through thermogravimetric analysis.

Thermogravimetric analysis (TGA) was carried out using Q600 (TA Instruments, New Castle, DE, USA) as shown in Figure 9 to obtain the fibre from the composite sample as the polymer was degraded using high temperature. Figure 10. SEM analysis showing fibre distribution with resin, along with voids between deposited shows the micrograph of the through-thickness sample which was stopped in the middle of 3D printing to observe the surface and the voids between the layers. Voids can be clearly seen between the layers, the fibres can also be seen, which are impregnated with the matrix. Additionally, in some areas, clusters of carbon fibre can be seen along with clearly visible voids. From Figure 11, which is at a magnification of 20 µm, it can be concluded that the average diameter of a single fibre is approximately equal to 8.2 µm. While Figure 12 shows the random orientation of the fibre that was analyzed. The through-thickness tension test is very sensitive to void content and fibre volume fraction. It must be noted that the through-thickness tensile strength is particularly sensitive to interply void content, which is different from that of void content throughout the laminate. Thermogravimetric analysis (TGA, Q600, TA Instruments, New Castle, DE, USA) (Figure 12) was carried out before SEM analysis to obtain the fibre from the composite sample as the polymer was degraded using high temperature. Figure 9 shows the TGA analysis that was performed at a rate of 10 °C/min to determine the degradation temperature, which was about 580 °C, and also to obtain fibre for SEM analysis.

## 5. Micro-Computed Tomography 

Micro-computed tomography (CT) was carried out to observe the fibre orientation and level of porosity inside the specimen using a SkyScan 1275 Bruker machine. The images were taken keeping the X-ray detector at 3 MP (1944 × 1536) with a 75 µm pixel size and a 20–100 kV range. A 10 W X-ray source with a <5 µm spot size at 4 W target power was used. Data Viewer, an imaging software of Bruker, was used to process the images. 

**Figure 13:**
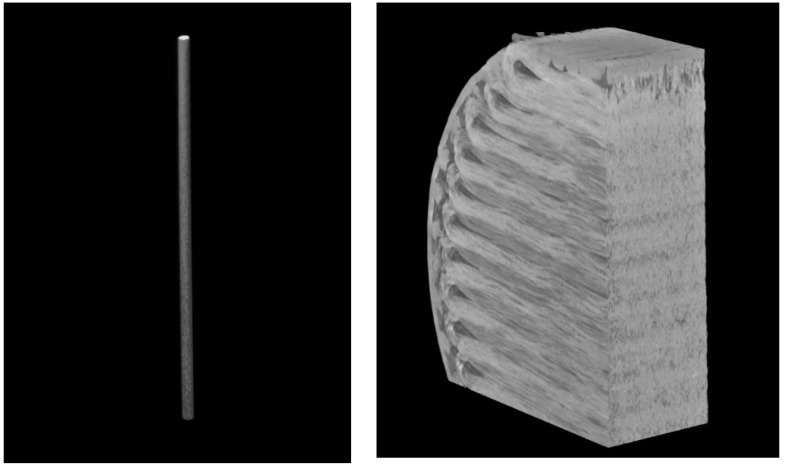
Micrograph of a single carbon fibre and cross-section of a through-thickness sample.

**Figure 14:**
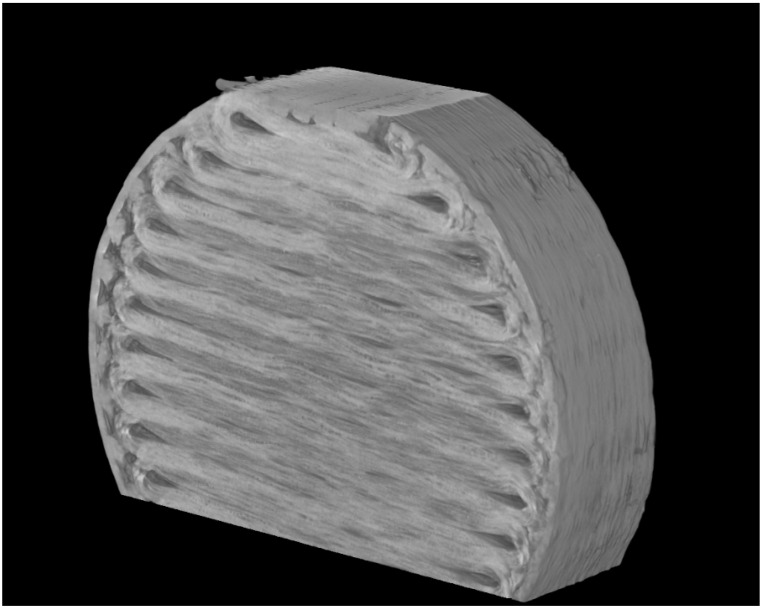
Micrograph of a through-thickness sample showing the layer path and the void distribution.

From the CT analysis of the single fibre, as shown in Figure 13, it can be seen that it exhibits a high degree of inhomogeneity which results in polymer-rich and fibre-rich regions, mostly due to the filament fabrication method, which may lead to premature failure of the materials. It is also worth noting that entrapped voids are present (evident) within the filament, which may also contribute to the formation of voids in the printed composites. Figure 14 shows that a lot of voids are present at the corner of the specimen as the Mark Forged Two 3D printer is not able to deposit the fibre in narrow regions, which is one of the limitations of the composite printer. It also increases the number of voids between the edge and fibre layers which causes the failure of the sample, as also observed in the literature by Liu et al. [17]. From the CT analysis, a void content of about 16% was observed in the composite samples, which is much higher as compared to composite laminate manufactured by traditional techniques, such as the void content of up to 5% for the vacuum-assisted resin transfer moulded method [18]. 

## 6. Conclusions and Future Work

Through-thickness tests were carried out to determine the mechanical properties of 3D printed samples after a successful set of experiments to measure both elastic modulus and strength. A maximum value for tensile strength of 12 MPa and an elastic modulus value of 2.94 GPa was measured for the circular specimen; for the rectangular specimen, a tensile strength of 7.8 MPa and an elastic modulus of 2.8 GPa were obtained. The maximum value of tensile strength that is obtained by other researchers is 7 MPa, while the elastic modulus has not been calculated so far, to the best of the author’s knowledge. From the microscopic examination, it was observed that some areas are resin rich, and some fibres were missing, which reduced the overall strength of the composite samples.

One of the conclusions that was noticed from these experiments is that the geometry had an effect and correlation on the strength in the z-direction. More work needs to be done in the future to minimize the printing defects, as well as to increase the amount of fibre contents. A poor fibre matrix interface was among the main causes of reduced mechanical performance. To have more accuracy for elastic modulus, strain gages must be mounted on each side of the specimen.

## Figures and Tables

**Table 1:** Dimension (mm) and failure load of 3D printed samples.

Specimen	Thickness (t)	Width(W)	Failure Load (N)
1	11.05	16.43	1309.12
2	11.06	16.27	1919.30
3	11.04	16.35	968.43
4	11.06	16.39	1209.40
5	10.06	16.37	1136.79
Average values	11.05	16.32	1228.79
